# Comparative Genomics of *Lactobacillus acidipiscis* ACA-DC 1533 Isolated From Traditional Greek Kopanisti Cheese Against Species Within the *Lactobacillus salivarius* Clade

**DOI:** 10.3389/fmicb.2018.01244

**Published:** 2018-06-11

**Authors:** Maria Kazou, Voula Alexandraki, Jochen Blom, Bruno Pot, Effie Tsakalidou, Konstantinos Papadimitriou

**Affiliations:** ^1^Laboratory of Dairy Research, Department of Food Science and Human Nutrition, Agricultural University of Athens, Athens, Greece; ^2^Bioinformatics and Systems Biology, Justus-Liebig-University Giessen, Giessen, Germany; ^3^Research Group of Industrial Microbiology and Food Biotechnology (IMDO), Department of Bioengineering Sciences (DBIT), Vrije Universiteit Brussel, Brussels, Belgium

**Keywords:** *Lactobacillus*, genome, pseudogene, motility, horizontal gene transfer, phage, probiotic, metabolism

## Abstract

*Lactobacillus acidipiscis* belongs to the *Lactobacillus salivarius* clade and it is found in a variety of fermented foods. Strain ACA-DC 1533 was isolated from traditional Greek Kopanisti cheese and among the available *L. acidipiscis* genomes it is the only one with a fully sequenced chromosome. *L. acidipiscis* strains exhibited a high degree of conservation at the genome level. Investigation of the distribution of prophages and Clustered Regularly Interspaced Short Palindromic Repeats (CRISPRs) among the three strains suggests the potential existence of lineages within the species. Based on the presence/absence patterns of these genomic traits, strain ACA-DC 1533 seems to be more related to strain JCM 10692^T^ than strain KCTC 13900. Interestingly, strains ACA-DC 1533 and JCM 10692^T^ which lack CRISPRs, carry two similar prophages. In contrast, strain KCTC 13900 seems to have acquired immunity to these prophages according to the sequences of spacers in its CRISPRs. Nonetheless, strain KCTC 13900 has a prophage that is absent from strains ACA-DC 1533 and JCM 10692^T^. Furthermore, comparative genomic analysis was performed among *L. acidipiscis* ACA-DC 1533, *L. salivarius* UCC118 and *Lactobacillus ruminis* ATCC 27782. The chromosomes of the three species lack long-range synteny. Important differences were also determined in the number of glycobiome related proteins, proteolytic enzymes, transporters, insertion sequences and regulatory proteins. Moreover, no obvious genomic traits supporting a probiotic potential of *L. acidipiscis* ACA-DC 1533 were detected when compared to the probiotic *L. salivarius* UCC118. However, the existence of more than one glycine-betaine transporter within the genome of ACA-DC 1533 may explain the ability of *L. acidipiscis* to grow in fermented foods containing high salt concentrations. Finally, *in silico* analysis of the *L. acidipiscis* ACA-DC 1533 genome revealed pathways that could underpin the production of major volatile compounds during the catabolism of amino acids that may contribute to the typical piquant flavors of Kopanisti cheese.

## Introduction

The genus *Lactobacillus* constitutes a diverse group of bacteria comprising more than 200 species and subspecies^1^ that are ubiquitous and frequently found in a variety of nutrient-rich ecological niches (Pot et al., 2014; Sun Z. et al., 2015). Lactobacilli produce lactic acid as the main end-product of carbohydrate fermentation allowing them to prevail in microbial ecosystems. This attribute along with their safety profile and their ability to shape organoleptic characteristics of the final product are the central reasons for their extensive use in artisanal or industrial food fermentations (Bernardeau et al., 2008; Sun Z. et al., 2015; Reginensi et al., 2016). Apart from food-related lactobacilli, the genus includes many commensals of the human, animal and plant microbiota (Cannon et al., 2005; Duar et al., 2017). The available genomes for *Lactobacillus* species and the close phylogenetic relationship among food- and host-related strains offer a wealth of information that underpin specialized mechanisms of bacterial adaptation to different environments (Sun Z. et al., 2015).

*Lactobacillus acidipiscis* is a salt-tolerant species originally isolated from fermented fish (Tanasupawat et al., 2000). The species has been also found in a variety of cheeses, i.e., Halloumi (Lawson et al., 2001; Naser et al., 2006; Kim et al., 2011), Cotija (Morales et al., 2011), Minas (Perin et al., 2017), and double cream cheese (Morales et al., 2011; Melgar-Lalanne et al., 2013), as well as in fermented fish (An et al., 2010; Tsuda et al., 2012; Thamacharoensuk et al., 2017), fermented meat (Nguyen et al., 2013), sake (Koyanagi et al., 2016), pickles (Arasu et al., 2015), grasses (Tohno et al., 2012; Khota et al., 2016), mulberry silage (Altaher et al., 2015), sweet paste (Mao et al., 2017), the traditional Chinese fermented vegetable Sichuan paocai, (Cao et al., 2017) and table olives (Randazzo et al., 2017). Moreover, *L. acidipiscis* has also been found in vinegar (Li P. et al., 2014) and soy sauce, where it is considered to be a spoiler (Tanasupawat et al., 2002; Cheng et al., 2014; Li N. et al., 2014). *L. acidipiscis* ACA-DC 1533 was isolated from traditional Greek Kopanisti cheese and along with *Lactobacillus rennini* were the dominant microbiota of this cheese (Asteri et al., 2009). Interestingly, both of them produced alcohols and carbonyl compounds most probably *via* amino acid catabolism that may contribute to the typical piquant flavors of Kopanisti cheese (Yvon and Rijnen, 2001; Asteri et al., 2009; Donnelly, 2016).

Phylogenetic analysis of *L. acidipiscis* places the bacterium in the *Lactobacillus salivarius* clade. The *L. salivarius* clade is the second largest group of lactobacilli with 27 recognized species following that of *Lactobacillus delbrueckii* (29 species; Pot et al., 2014). The *L. salivarius* clade consists mainly of commensal isolates and to a lesser degree of strains found in fermented foods (Cousin et al., 2015). Several strains belonging to the clade exhibit putative probiotic traits (Neville and O’Toole, 2010). Therefore, comparative genomics among members of the *L. salivarius* clade may reveal important aspects, such as niche adaptation, technological potential, and probiotic properties (Forde et al., 2011; Raftis et al., 2011; Sun Z. et al., 2015). So far, there are eight genomes with fully sequenced chromosomes in the *L. salivarius* clade publicly available in the NCBI database, i.e., six from *L. salivarius* (Claesson et al., 2006; Jimenez et al., 2010; Raftis et al., 2014; Chenoll et al., 2016), one from *Lactobacillus ruminis* (Forde et al., 2011) and one from *L. acidipiscis* (Kazou et al., 2017). Furthermore, *L. acidipiscis* JCM 10692^T^ and DSM 15836^T^ isolated from fermented fish as well as *L. acidipiscis* DSM 15353 and KCTC 13900 isolated from Halloumi cheese have been partially sequenced (Kim et al., 2011; Sun Z. et al., 2015). In fact, strains JCM 10692^T^ and DSM 15836^T^ are replicas of the same strain^2,3^ and the same applies for strains DSM 15353 and KCTC 13900^4,5^ .

The genome sequence of *L. acidipiscis* ACA-DC 1533 has been published (Kazou et al., 2017) and the current study aims to examine aspects of the evolution, physiology, metabolism and technological properties of the species according to the available *L. acidipiscis* genomes. Furthermore, we perform comparative genomics among the species with fully sequenced genomes in the *L. salivarius* clade to shed light to niche adaptation (host or food related, or both). Our analysis reveals technological properties of *L. acidipiscis* ACA-DC 1533 that may support the potential use of the isolate in food fermentations.

## Materials and Methods

### Chromosome-Plasmid Sequences and Annotations

Species/strains employed in phylogenetic analysis and comparative genomics are presented in **Supplementary Table S1**. All annotated sequences derived from RefSeq version 86 with the exception of plasmids pLAC2 and pLAC3 of *L. acidipiscis* ACA-DC 1533 that have not been included in RefSeq yet, so we used their GenBank/ENA versions (Kazou et al., 2017). In the table we present all relevant information to aid the reader assess whether differences or similarities in gene content among strains analyzed may be influenced by differences in sequencing technologies and/or tools used for sequence assembly and annotation.

### Phylogenetic Analysis

A whole genome phylogenetic tree based on the core genes among representative strains of all species in the *L. salivarius* clade using *L. acidipiscis* ACA-DC 1533 as the reference genome was constructed with the EDGAR software (Blom et al., 2009). It should be noted that whenever available, sequences of type strains were preferred. Core gene sets were aligned using MUSCLE, the individual alignments were concatenated and the resulting genome alignment was used as input for the construction of the phylogenetic tree with the neighbor-joining method as implemented in the PHYLIP package. *Weissella kandleri* DSM 20593^T^ and *Lactobacillus delbrueckii* subsp. *bulgaricus* ATCC 11842^T^ were used as outgroups.

### Comparative Genomic Analysis

To confirm the clonal relation among sequenced strains of *L. acidipiscis* as these are deduced from different databases, we used an ANI heat map as calculated with the EDGAR tool. The completeness of partial genome sequences of *L. acidipiscis* strains was assessed using the dBBQs (Wanchai et al., 2017). Preliminary evaluation of the presence of plasmids in the partially sequenced *L. acidipiscis* strains was performed with the r2cat tool (Husemann and Stoye, 2010), using as templates the three pLAC plasmid sequences of strain ACA-DC 1533. The circular map of *L. acidipiscis* ACA-DC 1533 was constructed by the DNAPlotter software (Carver et al., 2009). Pan/core-genome and singleton analysis were conducted with EDGAR. Comparison of the motility gene clusters among *L. acidipiscis* ACA-DC 1533 and KCTC 13900 as well as *Lactobacillus curvatus* NRIC 0822 was performed with the Easyfig comparison tool (Sullivan et al., 2011). The GenBank accession numbers for the motility operons of *L. acidipiscis* KCTC 13900 and *L. curvatus* NRIC 0822 are KM886858 and KM886863, respectively (Cousin et al., 2015). The EggNOG server version 4.5 was used for COG annotation (Huerta-Cepas et al., 2016). COG frequency heat maps with double hierarchical clustering were generated using the RStudio and the package “gplots”^6^. GIs, ISs, putative prophages, CRISPRs, RM systems, TA systems and putative antimicrobial peptides were predicted using the IslandViewer 4 web-based resource (Bertelli et al., 2017), the ISsaga platform (Varani et al., 2011), the PHASTER web server (Arndt et al., 2016), the CRISPRFinder web tool (Grissa et al., 2007), the REBASE database (Roberts et al., 2015), the TAfinder (Xie et al., 2018) and the BAGEL (van Heel et al., 2013), respectively. The glycobiome profile was investigated using the dbCAN (Yin et al., 2012) against the CAZy database (Lombard et al., 2014). Furthermore, transporters were determined using the TransportDB database (Elbourne et al., 2017). Pathways were assigned with the KEGG database (Kanehisa et al., 2016). Regulatory proteins including TCSs, TFs, and ODPs were detected with the P2RP web server (Barakat et al., 2013). Full-length chromosome alignments were created by progressiveMAUVE (Darling et al., 2010). Finally, the carbohydrate fermentation profile of *L. acidipiscis* ACA-DC 1533 was determined using API 50 CHL stripes (bioMérieux, Marcy-l’Etoile, France).

## Results and Discussion

### Whole Genome Phylogeny of the *L. salivarius* Clade

**FIGURE 1:**
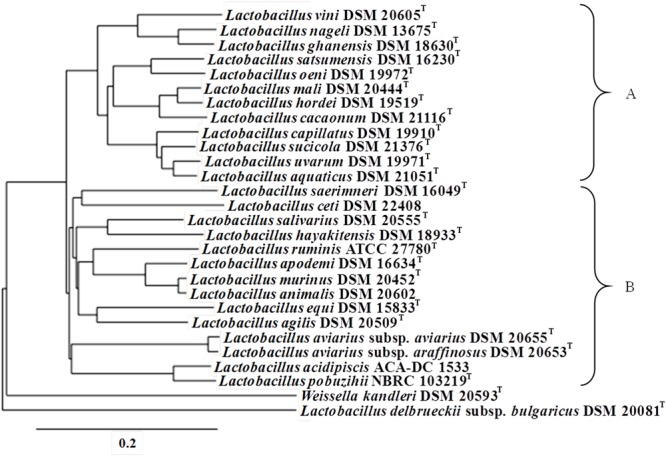
Whole genome phylogenetic tree for representative species of the *L. salivarius* clade. The tree was calculated based on the core-genome and it is presented as a cladogram. Species were separated in two main groups, namely A and B, as depicted in the figure. *Weissella kandleri* DSM 20593^T^ and *Lactobacillus delbrueckii* subsp. *bulgaricus* ATCC 11842^T^ were used as outgroups.

**FIGURE 2:**
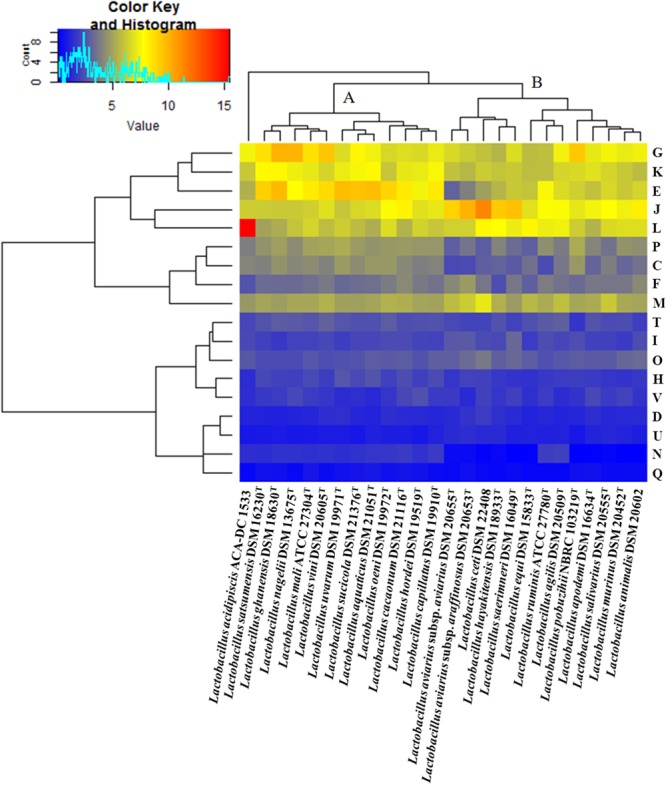
COG frequency heat map based on a two-dimensional hierarchical clustering. The horizontal axis shows the percentage frequency of proteins involved in each functional COG category and the vertical axis the representative strains of the species in the *L. salivarius* clade.

The phylogenetic relationship among the species of the *L. salivarius* clade was determined based on whole genome sequences. Analysis with the EDGAR software revealed two major clusters containing 12 and 14 species, respectively (**Figure 1**). *L. acidipiscis* was grouped together with *Lactobacillus pobuzihii* in a cluster, which also included *L. salivarius*. The strains employed in the phylogenetic analysis of the *L. salivarius* clade exhibited a pan genome of 13,470 genes, while the core-genome consisted of 349 genes. Moreover, proteins of the species belonging to the *L. salivarius* clade were distributed into various COG functional categories with a relatively distinct profile for each species. Interestingly, hierarchical clustering of the COG frequency heat map (**Figure 2**) revealed two clusters, which were very similar to the two clusters mentioned above that were obtained in the whole genome phylogenetic tree (**Figure 1**). It should be noted that *L. acidipiscis* ACA-DC 1533 was placed separately from these two clusters most probably due to an increased percentage of genes in the replication, recombination and repair (L) COG category. This difference could arise from a higher number of transposases in the ACA-DC 1533 genome but the number of transposases in the partial genomes employed during this analysis may be severely skewed. Nevertheless, *L. acidipiscis* also exhibited a higher number of transposases when compared to the complete genome sequences of *L. salivarius* and *L. ruminis* (please see below). Both whole genome phylogeny and COG analysis can be influenced by the partial nature of some of the sequences employed as well as differences in pipelines used for genome assembly and annotation. However, the whole genome phylogenetic tree is similar in the overall topology to the 16S rRNA phylogenetic tree of the entire *Lactobacillus* genus published by Pot et al. (2014) which is independent of genome completeness and annotation. The same applies when we compared our whole genome phylogenetic tree to the tree based on the concatenated amino acid sequences of 16 marker genes published by Sun Z. et al. (2015).

### General Genomic Features of *L. acidipiscis* Strains

To date, there are five sequenced strains of *L. acidipiscis*, i.e., ACA-DC 1533, KCTC 13900, DSM 15353, JCM 10692^T^ and DSM 15836^T^. As mentioned above, strains KCTC 13900 and DSM 15353 as well as JCM 10692^T^ and DSM 15836^T^ are replicas. Since this is not always obvious in the respective literature (Kim et al., 2011; Sun Z. et al., 2015), the relatedness among the two pairs of *L. acidipiscis* strains was also obtained by the ANI performed with EDGAR (**Supplementary Figure S1**). Results obtained confirmed the clonal relationship among the strains. To evaluate the level of completeness between the *L. acidipiscis* genomes in each of the two pairs of replica strains, we used the genome quality scores from the dBBQs based on the sequence completeness, the tRNA and rRNA score, as well as the number of essential genes predicted in the genome sequence (Wanchai et al., 2017). According to these results, strains KCTC 13900 and JCM 10692^T^ were found to be more complete than strains DSM 15353 and DSM 15836^T^, respectively (**Supplementary Table S2**). For this reason, strains KCTC 13900 and JCM 10692^T^ were employed for further analysis.

**FIGURE 3:**
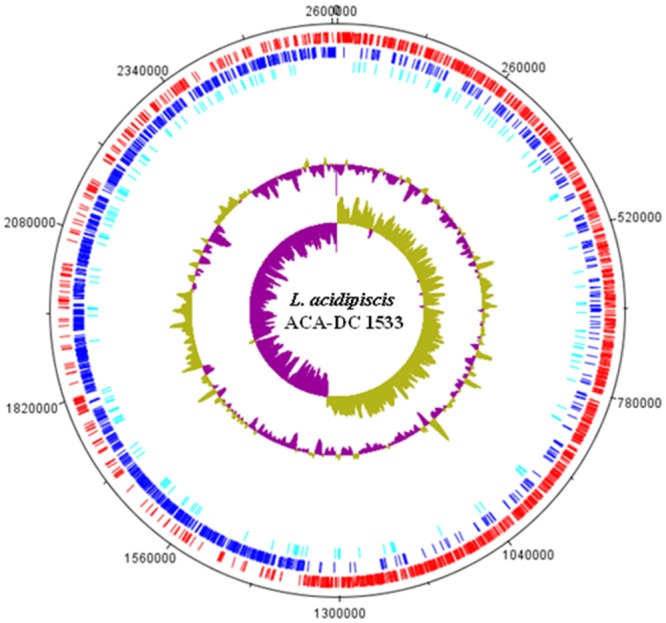
Circular map of the *L. acidipiscis* ACA-DC 1533 chromosome. Labeling from the outside to the inside circle, each ring contains information of the chromosome: CDSs on the forward strand (red); CDSs on the reverse strand (blue); Putative pseudogenes (cyan); tRNA genes (green); rRNA genes (orange); GC content; GC skew.

The characteristics of the *L. acidipiscis* ACA-DC 1533 genome were described previously (Asteri et al., 2010; Kazou et al., 2017). The complete chromosomal sequence of the strain was recently re-annotated in RefSeq revealing a total of 2,455 genes including 2,199 protein-coding genes and 172 potential pseudogenes mostly due to frame shifting and internal stop codons (**Figure 3**). Among pseudogenes, hypothetical proteins and mobile elements, such as ISs and transposases, were the most common (**Supplementary Table S3**). The genome also includes six rRNA operons distributed throughout the genome and 63 tRNA genes with the majority located around the five rRNA operons (data not shown).

**FIGURE 4:**
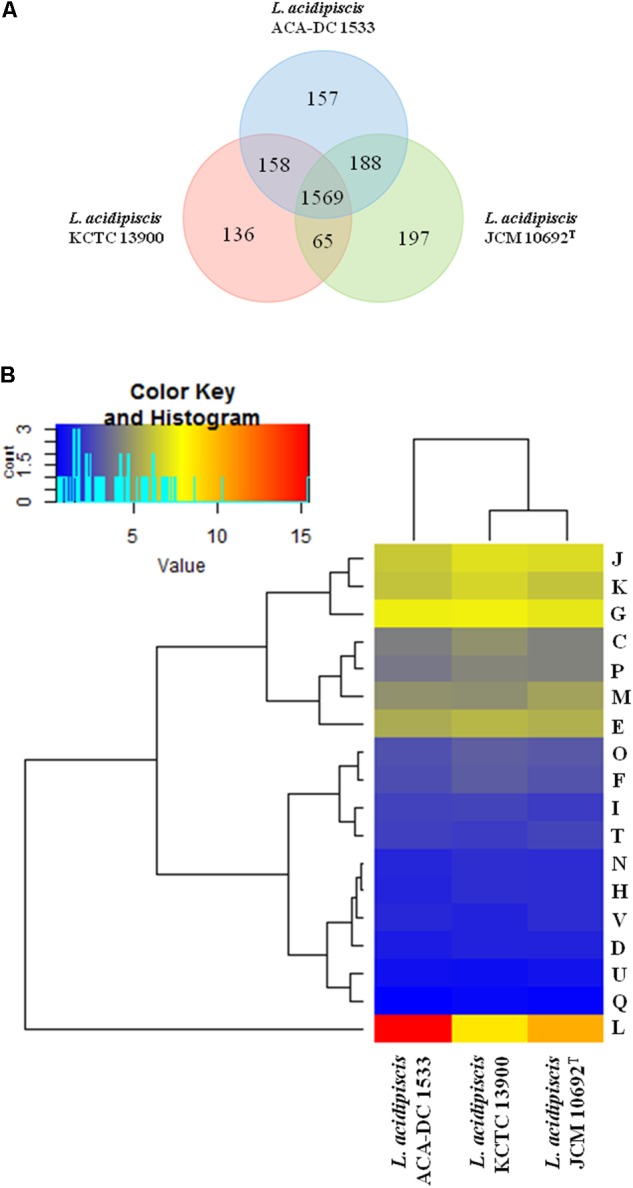
CDS Venn diagram of the three *L. acidipiscis* strains. In the intersection of the three strains we present their total core-genome, in the intersection of each pair of strains we present the corresponding core-genome and finally we present the singleton of each strain all calculated with the EDGAR tool **(A)**. COG frequency heat map based on a two-dimensional hierarchical clustering. The horizontal axis shows the three *L. acidipiscis* strains, namely ACA-DC 1533, KCTC 13900 and JCM 10692^T^ and the vertical axis the percentage frequency of proteins involved in each functional COG category **(B)**.

The additional *L. acidipiscis* assemblies of strains JCM 10692^T^ and KCTC 13900 are fragmented and thus do not allow the determination of their accurate chromosomal size as well as the evaluation of their plasmid content. Nevertheless, in these assemblies we could detect plasmid sequences after analysis with the r2cat tool, using as templates the three pLAC plasmid sequences (data not shown). Strain ACA-DC 1533 exhibits 2,288 protein-coding genes versus 2,126 and 1,969 for the JCM 10692^T^ and KCTC 13900 strains, respectively. Analysis with EDGAR revealed that the pan-genome consists of 2,722 genes, with 1,569 and 411 genes belonging to the core- and the dispensable genomes, respectively (**Figure 4A** and **Supplementary Tables S4A,B**). Furthermore, the analysis revealed that singletons represented approximately the 18% of the pan-genome. Strain JCM 10692^T^ carries the highest number of singletons (*n* = 197) followed by strains ACA-DC 1533 (*n* = 157) and KCTC 13900 (*n* = 136) (**Supplementary Table S4C**). However, such differences may not be readily explained given the differences in completeness among these genomes. We would like to mention that the total number of genes for each strain presented in **Figure 4A** is somewhat lower than the total number of genes annotated for the strain. The missing genes are genes that are not part of the 3-genome or 2-genome cores, but also do not appear in the strictly calculated singletons as they have some second-best BLAST hits, or non-reciprocal-BLAST hits or in general show some similarity to other genes in the dataset that rules them out as singletons as calculated by the EDGAR tool. The distribution of proteins into the COG functional categories is shown in a heat map for the three *L. acidipiscis* strains (**Figure 4B**). Despite their differences in completeness, the three genomes present very similar percentages in each of the COG categories. There was only one exception in replication, recombination and repair (L) category in which strain ACA-DC 1533 appears to have 15.4% compared to 10.3% and 8.6% for strains JCM 10692^T^ and KCTC 13900, respectively. As mentioned above, this higher percentage of proteins in the L category for strain ACA-DC 1533 was also evident in the comparison of all species within the *L. salivarius* clade (**Figure 2**). This difference may again reflect the fragmented nature of *L. acidipiscis* JCM 10692^T^ and KCTC 13900 genome assemblies.

**FIGURE 5:**
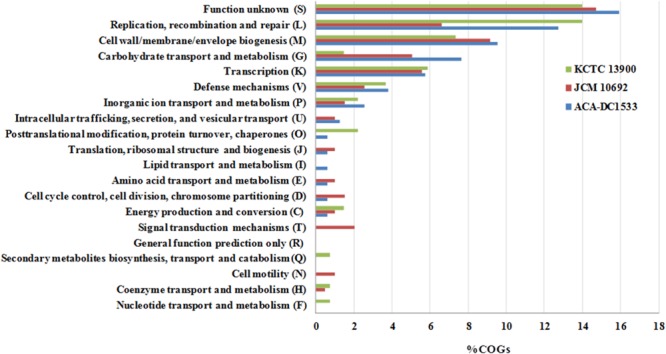
Distribution of singletons in COG functional categories of the three *L. acidipiscis* strains.

COG functional classification of the singletons is shown in **Figure 5**. We could find singletons of the three strains distributed in all COG categories with the majority associated with replication, recombination and repair (L), cell wall/membrane/envelope biogenesis (M), carbohydrate transport and metabolism (G) and transcription (K). The high prevalence of proteins in the L COG category appears again, this time in all three strains, especially strains ACA-DC 1533 and KCTC 13900. Strain JCM 10692^T^ appears to have approximately half the singletons in the L COG category, but this may be an artifact deriving from its partial sequence. It is unclear whether genes involved in information storage and processing might have technological implications. It could be suggested though, that the efficiency of central cellular mechanisms like those of the L, M, and K COG categories may provide the strain/species with a competitive advantage in a complex ecosystem. On the contrary, carbohydrate transport and metabolism can have a direct impact on the diversity of ecological niches in which the bacterium can grow.

### *In Silico* Evaluation of Motility of *L. acidipiscis* Strains

**FIGURE 6:**
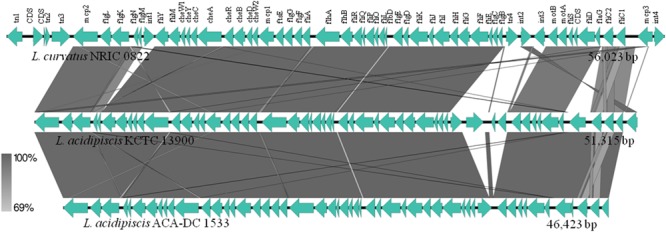
Sequence alignment among the motility gene clusters of *L. curvatus* NRIC 0822, *L. acidipiscis* KCTC 13900 and *L. acidipiscis* ACA-DC 1533. Gray shading corresponds to the % identity of the nucleotide sequences.

From a microbial ecology point of view, motile species may have competitive benefits against non-motile species, regarding e.g., niche colonization and biofilm formation (Neville et al., 2012). Currently, 16 motile *Lactobacillus* species have been recognized in the entire genus, all belonging to the *L. salivarius* clade with the exception of *L. curvatus*, which is a member of the *Lactobacillus sakei* clade (Cousin et al., 2015). Motility of *L. acidipiscis* has been recently described in strain KCTC 13900 revealing that the 54 proteins involved in flagellum regulation, synthesis, export and chemotaxis are organized in a single operon (Cousin et al., 2015). Annotation of ACA-DC 1533 identified 51 motility genes (LAC1533_RS09635-RS09885) producing a functional flagellar apparatus as also observed by *in vivo* experiments (data not shown). Core-genome analysis revealed that the motility operon is also present in strain JCM 10692^T^ and flanked by the same genes (**Supplementary Table S4B**). As shown in **Figure 6**, alignment of the motility operons of *L. curvatus* NRIC 0822 and *L. acidipiscis* strains KCTC 13900 and ACA-DC 1533 revealed that they are conserved.

### GIs Found in *L. acidipiscis* Genomes

HGT is one of the main processes responsible for genome evolution. Genomic fragments acquired by HGT events are characterized as GIs and may have a direct impact on the genome plasticity (Juhas et al., 2009). Here, we focused our analysis in the 13 GIs of the ACA-DC 1533 chromosome identified by the IslandViewer software tool (**Supplementary Figure S2**). Of note, GI 9 contains the genome’s array of ribosomal proteins (**Supplementary Table S5**). This is most probably a false positive result, as genes encoding ribosomal proteins have differences in sequence composition compared to regular protein coding genes (Fernández-Gómez et al., 2012) and are thus detected wrongfully by IslandViewer as part of a GI. For this reason, GI 9 was excluded from further analysis. The remaining 12 putative GIs contain a total of 229 genes and the respective lengths ranging from 4,677 to 36,954 bp. Many of these genes are involved in carbohydrate, lipid and amino acid metabolism as well as in membrane transport systems. According to the pan-genome analysis, GIs 3, 7, and 8 are unique for strain ACA-DC 1533 while GIs 1, 4, and 6 are common in all three *L. acidipiscis* strains, indicating acquisition early in the evolution of the species. It is interesting to note that GI 5 is present in strains ACA-DC 1533 and JCM 10692^T^ but absent in KCTC 13900. Other GIs are shared among the *L. acidipiscis* strains to a variable degree (**Supplementary Table S5**).

### Prophage Sequences, CRISPR-Cas Systems, RM Systems and TA Systems of *L. acidipiscis* Strains

PHASTER allowed the identification of one intact (1,228,777-1,272,253 bp, from now on called phage 1) and two incomplete prophage regions (1,575,825-1,586,105 bp and 1,802,666-1,830,756 bp) in the ACA-DC 1533 chromosome. Phage 1 contains 53 CDSs, most of which encode hypothetical proteins (approximately 41.5%). Furthermore, phage tail proteins, capsid proteins and *attL*/*attR* sites flanking the prophage DNA were also identified. Phage 1 is related to several prophages most of which can be found in *Lactobacillus* genomes. Strain JCM 10692^T^ carries a phage region similar to phage 1 (from now on called phage 2) sharing 30 out of 53 proteins (**Supplementary Table S6A**). Furthermore, strain KCTC 13900 seems to have an intact prophage region (from now on called phage 3) of 40.8 Kbp length related also to *Lactobacillus* phages (**Supplementary Table S6A**).

Three CRISPR sequences (i.e., CRISPR1, 2, and 3) were only identified in strain KCTC 13900 (**Supplementary Table S6B**). BLASTN analysis of all the spacers identified in these three CRISPR-Cas systems showed that several of them, namely spacers 9, 11, 13, 14, 19, 20, and 21 in CRISPR 1 and spacers 5, 14 and 21 in CRISPR 2 had hits in the *Lactobacillus plantarum* virulent phage phiJL-1. Moreover, spacers 22 and 26 in CRISPR 2 had hits in *L. salivarius* plasmids. Since *L. salivarius* strains carrying such plasmids are related to the host environment, this may suggest that *L. acidipiscis* has occupied this niche as well. Most importantly, spacers 1, 3, 5, 6, and 7 in CRISPR 1 and spacer 35 in CRISPR 2 had hits against phage 1 and/or phage 2 genes. Spacers in CRISPRs can reveal aspects of the evolutionary history of their host (Papadimitriou et al., 2014). Thus, it could be hypothesized that strain KCTC 13900 has also been exposed to phage 1 or phage 2 but it was able to acquire immunity through its CRISPR-Cas systems. Our findings may indicate that phages 1 or 2 are abundant in the ecological niches occupied by different *L. acidipiscis* strains or that, despite the different origins of isolation, the three *L. acidipiscis* strains were present in the same ecological niche sometime in the past. Moreover, the presence of phages 1 and 2 in the ACA-DC 1533 and JCM 10692^T^ genomes, respectively, corroborates with the lack of CRISPR systems in the two strains. However, the presence of prophages in the genomes of *L. acidipiscis* strains may protect them from superinfection by other phages or plasmids (Bondy-Denomy et al., 2016).

Bacterial defense mechanisms against foreign DNA include RM and TA systems (Darmon and Leach, 2014). Strain ACA-DC 1533 has a type I system that seems to be complete, as it contains the DNA-methyltransferase subunit M (LAC1533_RS04765), the specificity subunits S (LAC1533_RS04770) and R (LAC1533_RS04775), as well as a second type I system (LAC1533_RS01110-RS01130) possibly inactivated, since the restriction subunit R is a potential pseudogene (LAC1533_RS01130). According to the REBASE database, the strain also carries three putative type II RM systems (LAC1533_RS03065, LAC1533_RS05790 and LAC1533_RS08450-RS08455) and two type IV RM systems (LAC1533_RS02780 and LAC1533_RS04790) (**Supplementary Figure S3**). Plasmid pLAC3 also carries an *Ava*I RM system. Finally, we looked into TA systems. We concentrated our search on type II TA systems for which TAfinder prediction tool is available. In strain ACA-DC 1533 we found nine TA systems in the chromosome and one in the pLAC2 plasmid (**Supplementary Table S7**).

### Comparative Genomics of *L. acidipiscis* Against *L. salivarius* and *L. ruminis*

To further investigate the lifestyle and/or the technological traits of *L. acidipiscis* ACA-DC 1533, we performed comparative genomic analysis against *L. salivarius* UCC118 and *L. ruminis* ATCC 27782. *L. salivarius* UCC118 was chosen as the representative strain of the species since it is the first sequenced and presumably the best characterized strain of the clade (Harris et al., 2017). The comparison was performed initially at the chromosome level since the chromosomes of all three strains are completely sequenced. *L. salivarius* UCC118 was isolated from the human ileal-caecal region and comprises a chromosome of 1.8 Mbp and three plasmids, one of which is a megaplasmid of 242 Kbp (Claesson et al., 2006). *L. ruminis* ATCC 27782 isolated from the bovine rumen has a chromosome size of 2.1 Mbp with no plasmids (Forde et al., 2011). As mention above, *L. acidipiscis* ACA-DC 1533 has a chromosome of 2.6 Mbp, which is the largest among the three species. *L*. *acidipiscis* ACA-DC 1533 and *L. ruminis* ATCC 27782 exhibited the highest number of potential pseudogenes, i.e., 7.3 and 9.0%, respectively in contrast to the 2.8% of *L. salivarius* UCC188. However, other complete *L. salivarius* chromosomes exhibit a variable percentage of potential pseudogenes, up to 6.6% (**Supplementary Table S8**). Taking this observation into account, it seems that pseudogenes may not be constant among strains of the same species and thus the existence of only one complete chromosomal sequence for *L. acidipiscis* and *L. ruminis* are not enough to comment about their overall genome decay at the species level. Nevertheless, *L*. *acidipiscis* ACA-DC 1533 and *L. ruminis* ATCC 27782 appear to have undergone genome decay to an extent that is relatively restricted, at least when compared to the genome decay of highly specialized dairy lactobacilli like *L. delbrueckii* subsp. *bulgaricus* (van de Guchte et al., 2006).

**FIGURE 7:**
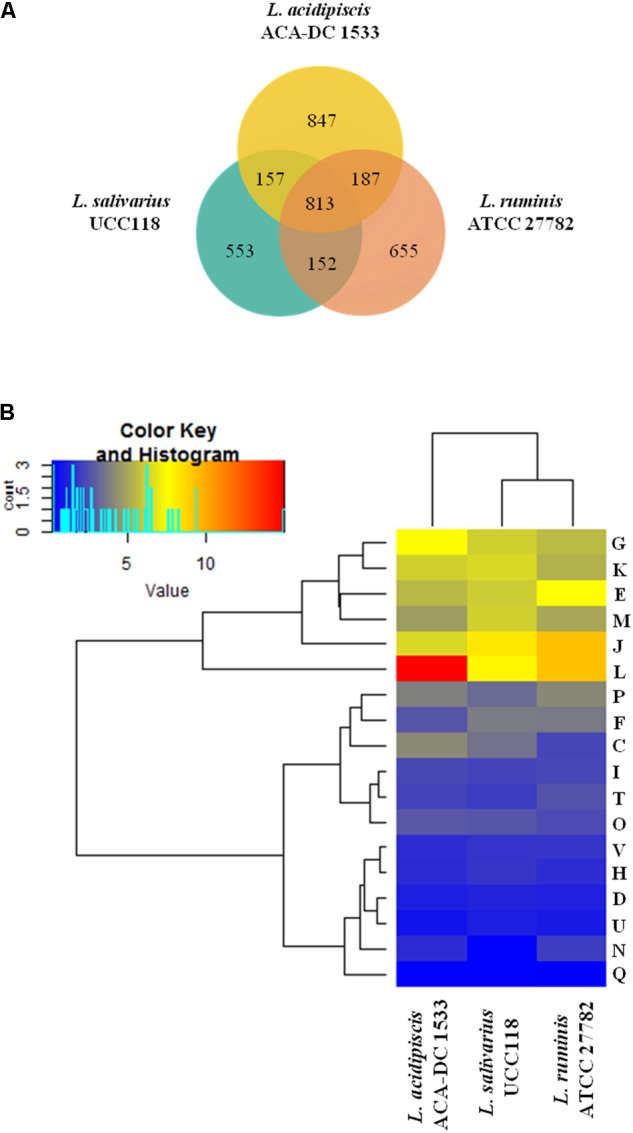
CDS Venn diagram of *L. acidipiscis* ACA-DC 1533, *L. salivarius* UCC118 and *L. ruminis* ATCC 17782. In the intersection of the three strains we present their total core-genome, in the intersection of each pair of strains we present the corresponding core-genome and finally we present the singleton of each strain all calculated with the EDGAR tool **(A)**. COG frequency heat map clustered hierarchically in two dimensions. The vertical axis shows the three genomes. The horizontal axis shows the % frequency of proteins involved in each functional COG category **(B)**.

Our analysis also revealed that the number of common proteins among the three species is 813, higher than that calculated for the entire *L. salivarius* clade as analyzed above (**Figure 7A** and **Supplementary Table S9A**). *L. acidipiscis* ACA-DC 1533 seems to carry the highest number of unique genes (*n* = 847) mostly encoding hypothetical proteins, transposases, ABC transporters, PEP-PTS and membrane transport proteins (**Supplementary Table S9B**). Similarly to **Figure 4A**, the total number of genes for each strain presented is somewhat lower than the total number of genes annotated for the strain since some genes cannot be assigned neither in the singletons nor in the 3-genome or 2-genome cores for the reason presented above. Furthermore, there is no extensive synteny among the three species as observed during full-length chromosome alignments created by progressiveMAUVE (**Supplementary Figure S4**). The analysis revealed a high number of LCBs with a quite short average length. Several studies based on comparative genomics among *Lactobacillus* species have established the genomic diversity of the *Lactobacillus* genus, which is higher compared to that of a typical bacterial family (Sun Z. et al., 2015; Martino et al., 2016).

The distribution of proteins into the COG functional categories for the three species is shown in **Figure 7B**. As expected, *L. acidipiscis* ACA-DC 1533 chromosome contained more proteins compared to *L. salivarius* UCC118 and *L. ruminis* ATCC 27782 in the L COG category owing to an inflated number of transposases and reverse transcriptases. Inspection of each of the two categories of gene products revealed that they may contain in some instances identical paralogs, but this is not always the case. The biological reason behind this observation is not clear. However, considering that both *L*. *salivarius* UCC118 and *L. ruminis* ATCC 27782 chromosomes are completely sequenced and that both *L. acidipiscis* ACA-DC 1533 and *L. ruminis* ATCC 27782 are annotated with the same pipeline in RefSeq, the possibility that this difference is some type of artifact is rather unlikely. Another obvious difference was the absence of proteins in the cell motility (N) COG category from the *L*. *salivarius* UCC118 chromosome. In all other COG categories, the distribution of proteins was at a comparable level among the three strains.

We also compared plasmid sequences of *L. acidipiscis* ACA-DC 1533 and *L. salivarius* UCC118. It has been shown for the latter that important housekeeping genes may be carried in its plasmids (Harris et al., 2017). In the case of *L. acidipiscis* plasmids most of the proteins were hypothetical. Nevertheless, we were able to identify some genes encoding proteins that may be important for the physiology, metabolism and/or the technological properties of the strain. For example, we determined the presence of carbohydrate and ion transporters (**Supplementary Table S10**), putative carbohydrate metabolizing enzymes (please see below), and an arsenate reductase. In addition and as mentioned above, plasmids of *L. acidipiscis* ACA-DC 1533 carry an *Ava*I RM system and a type II TA system (**Supplementary Table S7**).

### Glycobiome Analysis of *L. acidipiscis, L. salivarius*, and *L. ruminis*

The glycobiomes of *L. acidipiscis* ACA-DC 1533, *L. salivarius* UCC118 and *L. ruminis* ATCC 27782 were investigated using dbCAN. According to the analysis, *L. acidipiscis* ACA-DC 1533 had the largest glycobiome with 85 enzymes involved in carbohydrate metabolism, followed by *L. salivarius* UCC118 and *L. ruminis* ATCC 27782 with 78 and 68 enzymes, respectively (**Supplementary Table S11**). Among the 85 enzymes, 37 were identified as GHs, 21 as GTs, 13 as CEs and 14 as CBMs. Compared to the 37 GHs of *L. acidipiscis* ACA-DC 1533, *L. salivarius* UCC118 and *L. ruminis* ATCC 27782 contained 27 and 26 GHs, respectively. Among the GH families identified in the *L. acidipiscis* ACA-DC 1533, *L. salivarius* UCC118 and *L. ruminis* ATCC 27782 genomes, GH 13 was the most pronounced containing mainly enzymes with plant substrate specificity (Crost et al., 2013). Indeed, the carbohydrate fermentation profile of *L. acidipiscis* ACA-DC 1533 using the API 50 CHL stripes (**Supplementary Table S12**) and *L. salivarius* UCC118 (Li et al., 2006) showed that the two strains were able to ferment a number of carbohydrates of plant origin, i.e., L-arabinose, D-ribose, D-cellobiose, D-trehalose, D-glucose, D-fructose, D-mannitol, D-sorbitol, and D-saccharose. Furthermore, several GH families, namely GH 35, GH 38, GH 46, GH 70, and GH 76, were unique for the *L. acidipiscis* ACA-DC 1533 genome indicating that the bacterium presumably requires these enzymes in its ecological niche, which might be different to that of *L. salivarius* UCC118 and *L. ruminis* ATCC 27782. Interestingly, the presence of a beta-galactosidase (GH 35) and two 6-phospho-beta-galactosidase genes (GH 1) in the *L. acidipiscis* ACA-DC 1533 genome could probably be required for growth in milk. *L. acidipiscis* ACA-DC 1533 genome seems to contain also the highest number of CBM modules in family 50 compared to the *L. salivarius* UCC118 and *L. ruminis* ATCC 27782 genomes. CBM 50 modules are commonly found in bacterial lysins having a peptidoglycan binding function and a contribution to cell division (Visweswaran et al., 2013). Similarly to what has been reported previously for *L. salivarius* UCC118 (Harris et al., 2017) and according to our analysis, part of the glycobiome of both *L. salivarius* and *L. acidipiscis* ACA-DC 1533 resides in their plasmids. Specifically for *L. acidipiscis*, we found two GT 4 in plasmid pLAC2. It seems plausible to state that diversity of the plasmid glycobiome in strains of *L. salivarius* is significantly more rich than that of *L. acidipiscis* perhaps due to the presence of the megaplasmid. Moreover, analysis using the TransportDB database identified 47 potential sugar specific PTS transport proteins in the *L. acidipiscis* ACA-DC 1533 genome (3 on pLAC2) and 25 and 16 potential PTS transport proteins for *L. salivarius* UCC118 and *L. ruminis* ATCC 27782 genomes, respectively (**Supplementary Table S10**).

### Proteolytic System of *L. acidipiscis, L. salivarius*, and *L. ruminis*

The proteolytic system of lactic acid bacteria consists of cell-wall bound proteinases, which initiate the degradation of caseins, peptide and amino acid transport systems and a pool of intracellular peptidases, which further degrade the peptides to shorter peptides and free amino acids (Liu et al., 2010). The proteolytic system of the three *L. acidipiscis* strains, *L. salivarius* UCC118 and *L. ruminis* ATCC 27782 was investigated according to the scheme of Liu and co-workers (Liu et al., 2010) (**Supplementary Table S13**). The cell-wall bound proteinase (PrtP), the aminopeptidase A (PepA), the endopeptidases PepE/PepG and the proline peptidase PepL were missing from all strains. It is worth mentioning that PrtP gene is intact in plasmid pR1 of *L. salivarius* strain Ren (Sun E. et al., 2015). The rest of the peptidases were found in up to three copies per genome. Furthermore, *L. acidipiscis* ACA-DC 1533 and *L. ruminis* ATCC 27782 carried one oligopeptide ABC transport system (Opp), which was missing from the *L. salivarius* UCC118 genome. Interestingly, the Opp operon is present in *L. acidipiscis* ACA-DC 1533 and JCM 10692^T^ but absent in KCTC 13900. On the contrary, a di/tripeptide ABC transport system (Dpp) and a DtpT transporter of di- and tri-peptides were found in the three species (including all three *L. acidipiscis* strains). However, it is worth noting that the DppD protein of *L. acidipiscis* KCTC 13900 is a potential pseudogene inactivating the entire Dpp system which deserves further investigation. Moreover, *L. acidipiscis* ACA-DC 1533 chromosome seems to contain 17 amino acid ABC transport proteins, while *L. salivarius* UCC118 and *L. ruminis* ATCC 27782 chromosomes only 11 and 10, respectively. Even though the five *Lactobacillus* chromosomes and/or genomes carry a number of peptide and amino acid transporters as well as several intracellular peptidases, the absence of PrtP indicates that the strains may not directly hydrolyze large protein molecules, but they may take advantage of peptides and free amino acids already available in their ecological niche.

### Miscellaneous Genomic Features Deriving From the Comparison Among *L. acidipiscis* ACA-DC 1533, *L. salivarius* UCC118, and *L. ruminis* ATCC 27782

We also focused our analysis to IS elements that may contribute in bacterial genome evolution, to transport proteins which allow the transport of the substances in and out of the cell, as well as to RPs that control gene expression.

IS elements of *L. salivarius* UCC118 and *L. ruminis* ATCC 27782 have been previously identified (Claesson et al., 2006) but we have updated the analysis using the latest version of ISsaga and the most recent annotation files for the two strains. In the chromosomes of *L. acidipiscis* ACA-DC 1533, *L. salivarius* UCC118 and *L. ruminis* ATCC 27782, a total of 53, 10 and 30 IS elements were predicted with ISsaga, respectively (**Supplementary Table S14**). The higher number of IS elements in the chromosome of *L. acidipiscis* ACA-DC 1533 may suggest a higher potential for genome plasticity compared to the *L. salivarius* UCC118 and *L. ruminis* ATCC 27782 chromosome. The majority of IS elements in the *L. acidipiscis* ACA-DC 1533 chromosome belong to the ISL3 and IS982 families which were also previously identified in food related lactobacilli like *Lactobacillus delbrueckii* subsp. *bulgaricus* and *Lactobacillus helveticus*, respectively (Germond et al., 1995; Callanan et al., 2005).

Furthermore, the *L. acidipiscis* ACA-DC 1533 genome contains 287 transport proteins compared to 240 and 238 of *L. salivarius* UCC118 and *L. ruminis* ATCC 27782 genomes, respectively. They mainly belong to the ABC superfamily and to the MFS (**Supplementary Table S10**). Additional analysis of the *L. acidipiscis* ACA-DC 1533 genome revealed 17 potential glycine/betaine transport proteins organized in at least five distinct genomic loci. The glycine/betaine transport system may be necessary to overcome osmotic stress since *L. acidipiscis* is a salt-tolerant species owning strains able to grow in the presence of even 12% NaCl (our unpublished results; Tanasupawat et al., 2000; Romeo et al., 2003; Pot et al., 2014).

RPs include TCSs and TFs. TCSs are the most abundant phosphorylation-dependent signal transduction systems in prokaryotes and typically comprise a membrane-bound HK and a RR (Barakat et al., 2013). On the other hand, TFs contain TRs, OCSs, RRs and SFs. Analysis of *L. acidipiscis* ACA-DC 1533 and *L. salivarius* UCC118 identified six HKs and seven RRs for both strains. Analysis of *L. ruminis* ATCC 27782 chromosome revealed seven HKs and 10 RRs. Furthermore, the *L. acidipiscis* ACA-DC 1533 chromosome contained the highest number of TFs among the three strains analyzed, including 68 TRs, 28 OCSs, five RRs, six SFs and 19 ODPs, most of which were unclassified (**Supplementary Table S15**). The higher number of TFs in the *L. acidipiscis* compared to the other two species may suggest a more intricate regulation of gene expression and perhaps an increased interaction with the environment.

### Assessing the Probiotic and Technological Properties of *L. acidipiscis* ACA-DC 1533

Initially, we investigated the probiotic potential of *L. acidipiscis* ACA-DC 1533 based on the available information for *L. salivarius* UCC118 which has been extensively studied as a probiotic strain (Neville and O’Toole, 2010). The *L. salivarius* UCC118 genome contains a bile-salt hydrolase (Claesson et al., 2006) and two EPS clusters associated with the strain’s probiotic activity (Harris et al., 2017). These traits were absent from the *L. acidipiscis* ACA-DC 1533 genome. In addition, proteins that may play a role in the interaction of *L. salivarius* UCC118 with the host, may include mucus-, collagen-, salivary agglutinin- and epithelial-binding proteins, as well as enterococcal surface proteins (van Pijkeren et al., 2006; O’Shea et al., 2012). All these proteins are sortase-dependent surface proteins which were either absent from the *L. acidipiscis* ACA-DC 1533 genome or were characterized as potential pseudogenes. The only exception identified was a fibrinogen/fibronectin-binding protein, similar to that of *L. salivarius* UCC118 (Collins et al., 2012) that was also present in the *L. acidipiscis* ACA-DC 1533 genome. Furthermore, analysis of the *L. acidipiscis* ACA-DC 1533 genome with the BAGEL tool did not predict any bacteriocin gene, in contrast to the *L. salivarius* UCC118 genome, which produces the two-component class II bacteriocin Abp118 (Flynn et al., 2002). BAGEL also predicted in *L. acidipiscis* JCM 10692^T^ three potential structural genes coding for pediocin, sakacin P and carnocin like bacteriocins (the last being a potential pseudogene) and some accessory genes (e.g., immunity, transfer, and maturation) and further experimental testing for their production needs to be performed.

**FIGURE 8:**
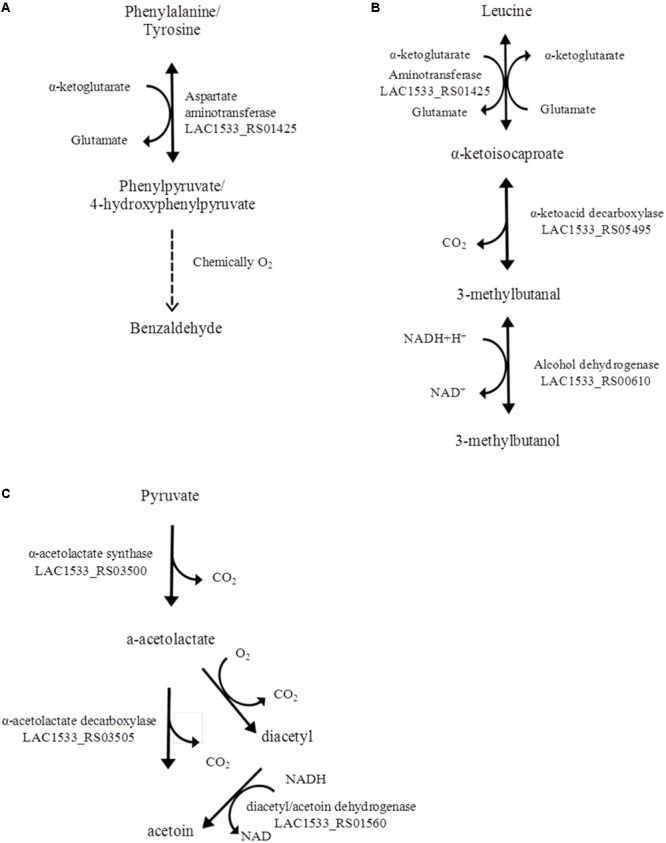
Catabolic pathways of the aromatic amino acids phenylalanine and tyrosine for the production of benzaldehyde **(A)**. Catabolic pathway of the branched chain amino acid leucine for the production of 3-methylbutanol **(B)**. Diacetyl and acetoin synthesis from pyruvate **(C)**.

We then investigated aspects of the technological potential of *L. acidipiscis* ACA-DC 1533 taking into account that Asteri and co-workers showed that the major volatile/flavor metabolites produced by this strain when grown in RSM and MRS, were 3-methylbutanal, 3-methylbutanol, benzaldehyde and acetoin (Asteri et al., 2009). The majority of the aforementioned metabolites produced by *L. acidipiscis* ACA-DC 1533 are degradation products of amino acids (**Figure 8**). In particular, benzaldehyde can be formed from two aromatic amino acids, namely phenylalanine and tyrosine, using an enzymatic and a non-enzymatic step (Nierop Groot and de Bont, 1998; Fernandez and Zuniga, 2006). Moreover, 3-methylbutanal and 3-methylbutanol are catabolic products of the branched-chain amino acid leucine (Fernandez and Zuniga, 2006). The α-ketoacid decarboxylase and the alcohol dehydrogenase involved in the leucine catabolism pathway were found to be present in the three *L. acidipiscis* genomes but absent from *L. salivarius* UCC118 and *L. ruminis* ATCC 27782. On the contrary, aspartate aminotransferase, which catalyzes the transamination of phenylalanine and tyrosine, was present in all the *Lactobacillus* genomes analyzed. Many studies have been shown that the amino acid degradation products, especially those deriving from the branched-chain, aromatic and sulfur-containing amino acids, are regarded as significant flavor compounds in several cheese varieties (Ardö, 2006; Liu et al., 2008; Afzal et al., 2017). Furthermore, acetoin, which was produced by *L. acidipiscis* ACA-DC 1533, can be formed from pyruvate using two alternative pathways. Pyruvate, which derives from glycolysis, is converted into a-acetolactate by α-acetolactate synthase (LAC1533_RS03500). α-Acetolactate is then catabolized either to acetoin by α-acetolactate decarboxylase (LAC1533_RS03505) or to diacetyl in the presence of oxygen. Finally, diacetyl/acetoin dehydrogenase (LAC1533_RS01560) catalyzes the conversion of diacetyl to acetoin (Celinska and Grajek, 2009). It should be mentioned that diacetyl was not detected as a volatile metabolite of *L. acidipiscis* ACA-DC 1533 in the work of Asteri et al. (2009). However, the presence of diacetyl/acetoin dehydrogenase in the ACA-DC 1533 genome could probably mean that by the time of sampling diacetyl was fully converted into acetoin. Given that *L. acidipiscis* ACA-DC 1533, along with *L. rennini*, were the only species found in Kopanisti cheese, the production of the above mentioned metabolites by *L. acidipiscis* ACA-DC 1533 *via* amino acid catabolism may contribute to the characteristic piquant flavor of Kopanisti cheese (Yvon and Rijnen, 2001; Asteri et al., 2009; Donnelly, 2016).

## Conclusion

The *L. salivarius* clade includes mainly commensal species and it has been suggested that several strains may have probiotic properties (Neville and O’Toole, 2010). In this study, we analyzed the available genomes of *L. acidipiscis*, a species within the *L. salivarius* clade that until today has mainly been isolated from fermented foods of dairy or other origin. We wanted to examine whether *L. acidipiscis* is also a commensal that is transferred to the ecosystem of fermented foods accidentally from the host. Furthermore, we wanted to investigate the probiotic and/or the technological potential of the species. We sequenced the genome of strain ACA-DC 1533, originally isolated from Kopanisti, a traditional spread-type cheese that is highly salted and particularly piquant (Kazou et al., 2017). Our investigation suggested that *L. acidipiscis* has a relatively large genome compared to other species of the *L. salivarius* clade (at least those of *L. salivarius* and *L. ruminis*) with a relatively restricted percentage of pseudogenes. These findings along with the observation that *L. acidipiscis* possesses a high number of glycobiome enzymes may indicate an ability to occupy versatile environments and has not been evolved toward a specific ecological niche. Perhaps, adaptation to a nutrient-rich niche would have been more consistent with a smaller genome size, more typically like that of *L. salivarius*. Interestingly, *L. acidipiscis* strains ACA-DC 1533 and JCM 10692^T^ appear to be more related compared to strain KCTC 13900 based on the presence/absence distribution of a number of genetic traits like prophages and CRISPRs. *L. acidipiscis* ACA-DC 1533 does not seem to present any evident probiotic trait at the genomic level. Besides the absence of several genes that have been related to probiotic properties of *L. salivarius* UCC118 preliminary experiments with strain *L. acidipiscis* ACA-DC 1533 on human PBMCs did not reveal an increased interleucine-10/interleucine-12 ratio, indicative of a potential to stimulate a Treg response (our unpublished results). Some probiotic properties have been suggested for specific *L. acidipiscis* strains like an antiproliferative effect against Caco-2 cells (Thamacharoensuk et al., 2017) or the improvement of feed conversion efficiency in broiler chickens (Altaher et al., 2015). Thus, further *in silico* and/or experimental assessment of the probiotic properties of *L. acidipiscis* may be required. In addition, *L. acidipiscis* is able to grow in the dairy environment since it can ferment lactose, it possesses a complete proteolytic system for the degradation of milk proteins (without carrying a cell-envelope proteinase) and it can produce volatile compounds during the catabolism of amino acids that may contribute to the flavor of the final product. Intriguingly, *L. acidipiscis* is also considered a spoiler in vinegar and soy sauce. For this reason, technological steps to prohibit its growth in certain fermented foods need to be devised. Further research is needed with different species of the *L. salivarius* clade like the newly sequenced *Lactobacillus agilis* to better appreciate the mechanisms underlining the adaptation to the host and/or the food environment. Sequencing of more strains/species would provide invaluable information about the ecology of this important clade within the *Lactobacillus* genus.

## Author Contributions

MK and VA performed genome analysis and participated in the writing of the manuscript. JB and BP performed genome analysis. ET conceived the project and participated in the writing of the manuscript. KP conceived the project, performed genome analysis, and participated in the writing of the manuscript. All authors read and approved the final manuscript.

## Conflict of Interest Statement

The authors declare that the research was conducted in the absence of any commercial or financial relationships that could be construed as a potential conflict of interest.

## Footnotes

1 http://www.bacterio.net/lactobacillus.html

2 http://www.jcm.riken.jp/cgi-bin/jcm/jcm_number?JCM=10692

3 https://www.dsmz.de/catalogues/details/culture/DSM-15836.html

4 https://www.dsmz.de/catalogues/details/culture/DSM-15353.html

5 http://kctc.kribb.re.kr/En/jsearch/j_sview.aspx?sn=13900

6 http://www.rstudio.org

## Supplementary Material

FIGURE S1 Heat map of ANI values among the five sequenced *L. acidipiscis* strains.

FIGURE S2 Circular map of the *L. acidipiscis* ACA-DC 1533 chromosome as generated by IslandViewer 4. Highlighted regions correspond to GIs. GIs are colored within the circular map according to the prediction method used: GIs in orange were predicted with SIGI-HMM, GIs in blue with IslandPath-DIMOB and the integrated GIs are presented on the periphery in red. The black line plot corresponds to the GC content (%) of the chromosomal sequence.

FIGURE S3 Circular map of the *L. acidipiscis* ACA-DC 1533 chromosome highlighting the predicted RM systems by the REBASE database. The symbols are color coded as indicated at the bottom of the figure.

FIGURE S4 Chromosome alignments among the *L. acidipiscis* ACA-DC 1533, *L. salivarius* UCC118 and *L. ruminis* ATCC 27782 strains generated by progressiveMAUVE. Locally collinear blocks (LCBs) of conserved sequences are presented by the same color (white corresponds to the strain-specific regions).

TABLE S1 General information of the strains analyzed in this study.

TABLE S2 Level of completeness among the four *L. acidipiscis* strains as calculated by dBBQs.

TABLE S3 Pseudogenes identified in the *L. acidipiscis* ACA-DC 1533 chromosome.

TABLE S4 **(A)** Pan-genome analysis among the three *L. acidipiscis* strains calculated with the EDGAR software. **(B)** Core-genome among the three *L. acidipiscis* strains calculated with the EDGAR software. **(C)** Singletons of the three *L. acidipiscis* strains calculated with the EDGAR software.

TABLE S5 Genes within genomic islands of *L. acidipiscis* ACA-DC 1533. Common genes within the rest of *L. acidipiscis* strains were predicted from the pan-genome analysis with the EDGAR software.

TABLE S6 **(A)** Prophage regions identified in the three *L. acidipiscis* strains using the PHASTER software. **(B)** CRISPR systems identified in the *L. acidipiscis* KCTC 13900 genome using the CRISPRFinder tool.

TABLE S7 TA systems predicted in the chromosome and pLAC2 plasmid of *L. acidipiscis* ACA-DC 1533 genome.

TABLE S8 Percentage of potential pseudogenes identified in the chromosomes of the completed sequenced genomes of *L. salivarius* clade.

TABLE S9 **(A)** Core-genome among the *L. acidipiscis* ACA-DC 1533, *L. salivarius* UCC118 and *L. ruminis* ATCC 27782 chromosomes as calculated with the EDGAR software. **(B)** Singletons among the *L. acidipiscis* ACA-DC 1533, *L. salivarius* UCC118 and *L. ruminis* ATCC 27782 chromosomes as calculated with the EDGAR software.

TABLE S10 Transporters of *L. acidipiscis* ACA-DC 1533 chromosome and pLAC2 plasmid identified by TransportDB.

TABLE S11 Putative enzymes involved in carbohydrate metabolism of *L. acidipiscis* ACA-DC 1533, *L. salivarius* UCC118, and *L. ruminis* ATCC 27782 chromosomes.

TABLE S12 Acid production by *L. acidipiscis* ACA-DC 1533 using API 50CHL stripes.

TABLE S13 The proteolytic system of *L. acidipiscis, L. salivarius*, and *L. ruminis* chromosomes and/or genomes.

TABLE S14 Insertion sequences (ISs) identified among the *L. acidipiscis* ACA-DC 1533, *L. salivarius* UCC118, and *L. ruminis* ATCC 27782 chromosomes using the ISsaga platform.

TABLE S15 Regulatory proteins identified in the chromosomes of *L. acidipiscis* ACA-DC 1533, *L. salivarius* UCC118, and *L. ruminis* ATCC 27782 using P2RP web server.

## Abbreviations

ABC: ATP-binding cassette
ANI: average nucleotide identity
CAZy: Carbohydrate Active EnZymes
CBMs: Carbohydrate-Binding Modules
CEs: Carbohydrate Esterases
COG: Clusters of Orthologous Groups
CRISPRs: Clustered Regularly Interspaced Short Palindromic Repeats
dBBQs: dataBase of Bacterial Quality scores
dbCAN: DataBase for automated Carbohydrate-active enzyme Annotation
EPS: Exopolysaccharide
GHs: Glycoside Hydrolases
GIs: Genomic islands
GTs: Glycosyl Transferases
HGT: Horizontal Gene Transfer
HK: Histidine Kinase
ISs: insertion sequences
KEGG: Kyoto Encyclopedia of Genes and Genomes
LCBs: locally collinear blocks
MFS: major facilitator superfamily
OCSs: One-component Systems
ODPs: Other DNA-binding Proteins
PBMCs: peripheral blood mononuclear cells
PEP-PTS: Phosphoenolpyruvate Phosphotransferase System
P2RP: Predicted Prokaryotic Regulatory Proteins
r2cat: Related Reference Contig Arrangement
RM: Restriction-Modification
RPs: Regulatory Proteins
RR: Response Regulator
RSM: reconstituted skim milk
SFs: Sigma Factors
TA: toxin-antitoxin
TCSs: Two-component Systems
TFs: Transcription Factors
TRs: Transcriptional Regulators
