# Supplementary figures and images for: Comparative Genomics of Lactobacillus acidipiscis ACA-DC 1533 Isolated From Traditional Greek Kopanisti Cheese Against Species Within the Lactobacillus salivarius Clade
Source: Front Microbiol. 2018 Jun 11;9:1244. doi: 10.3389/fmicb.2018.01244 (PMC6004923; doi:10.3389/fmicb.2018.01244)

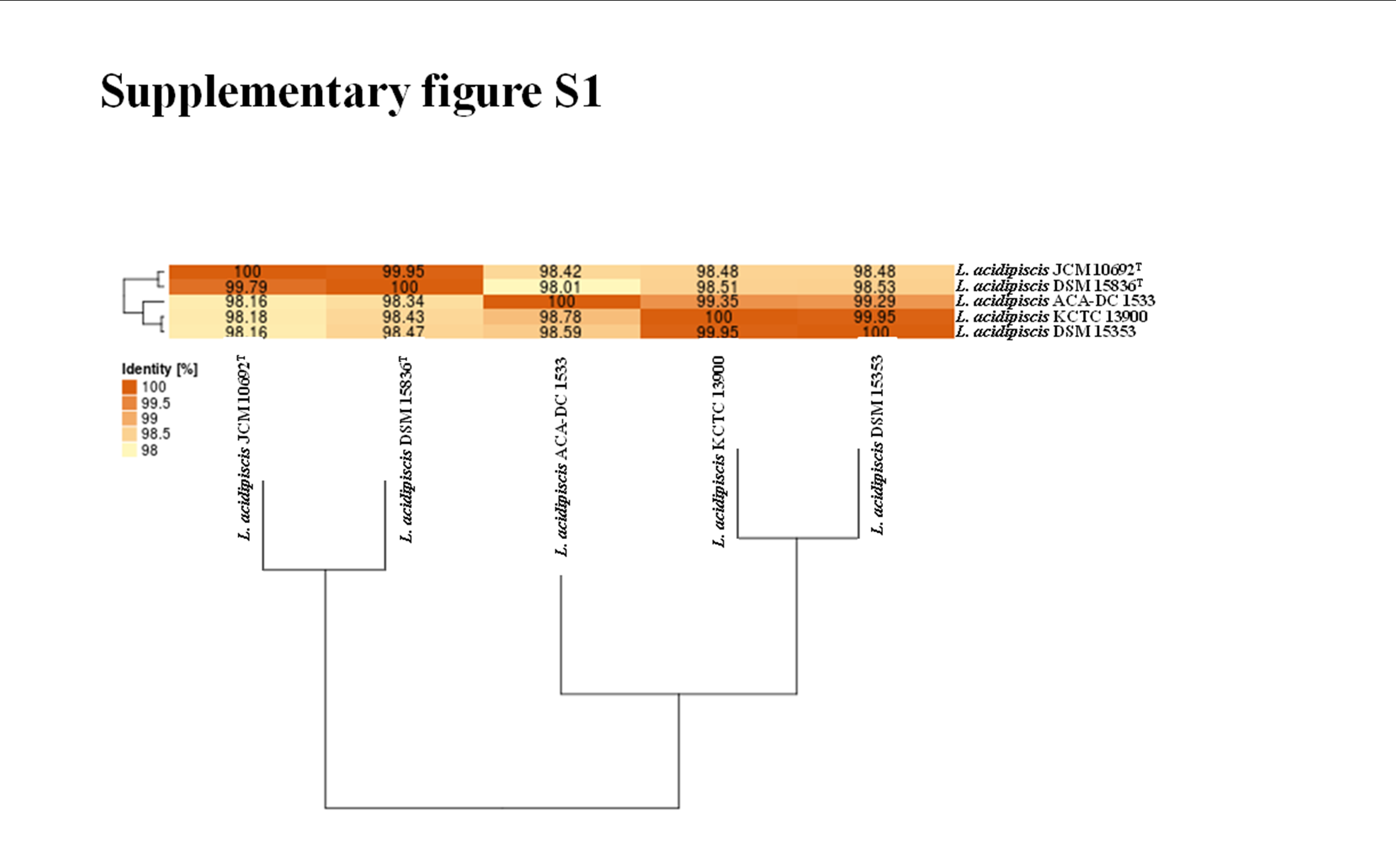

Supplement: FIGURE S1 — Heat map of ANI values among the five sequenced L. acidipiscis strains. [file Image_1.tif]

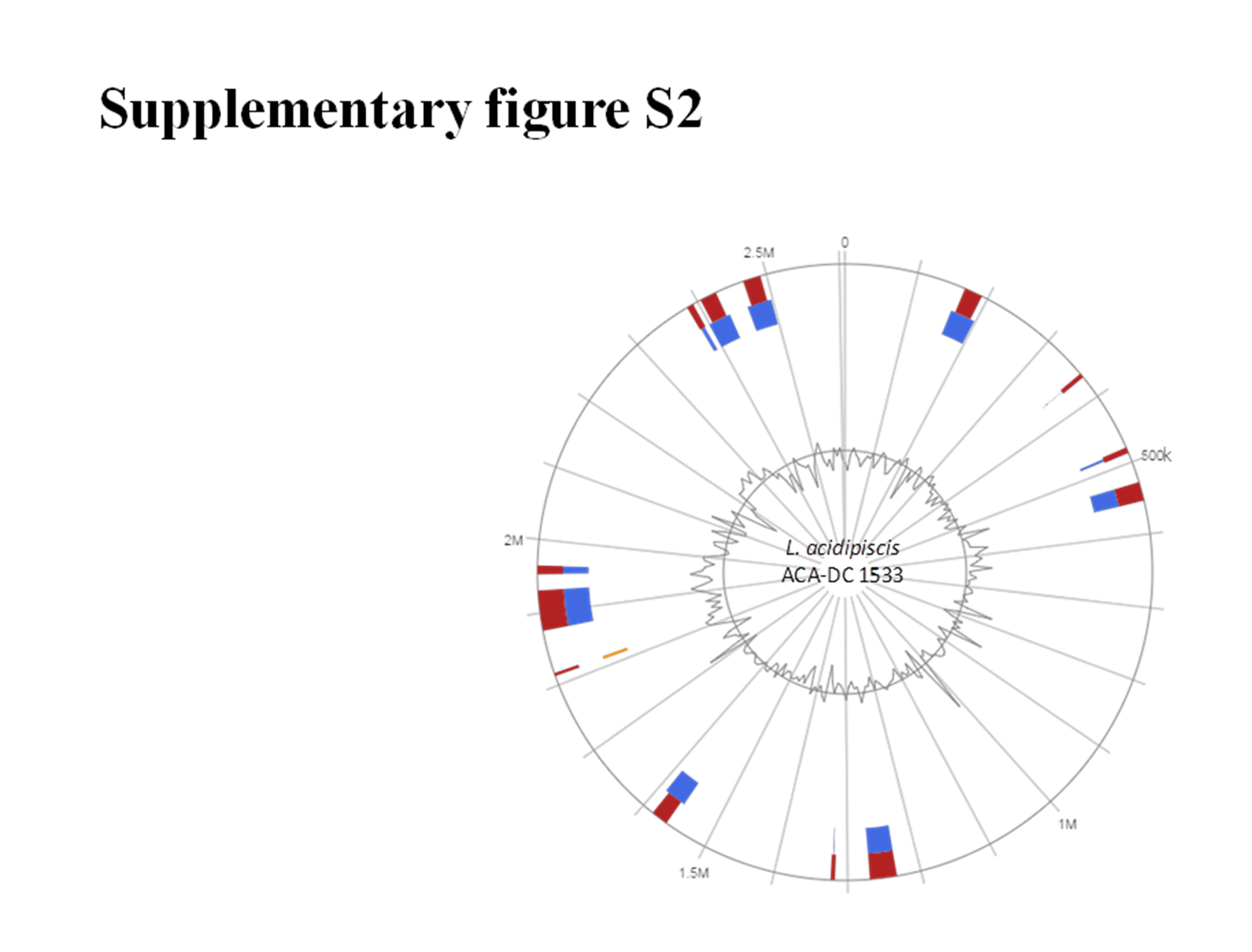

Supplement: FIGURE S2 — Circular map of the L. acidipiscis ACA-DC 1533 chromosome as generated by IslandViewer 4. Highlighted regions correspond to GIs. GIs are colored within the circular map according to the prediction method used: GIs in orange were predicted with SIGI-HMM, GIs in blue with IslandPath-DIMOB and the integrated GIs are presented on the periphery in red. The black line plot corresponds to the GC content (%) of the chromosomal sequence. [file Image_2.tif]

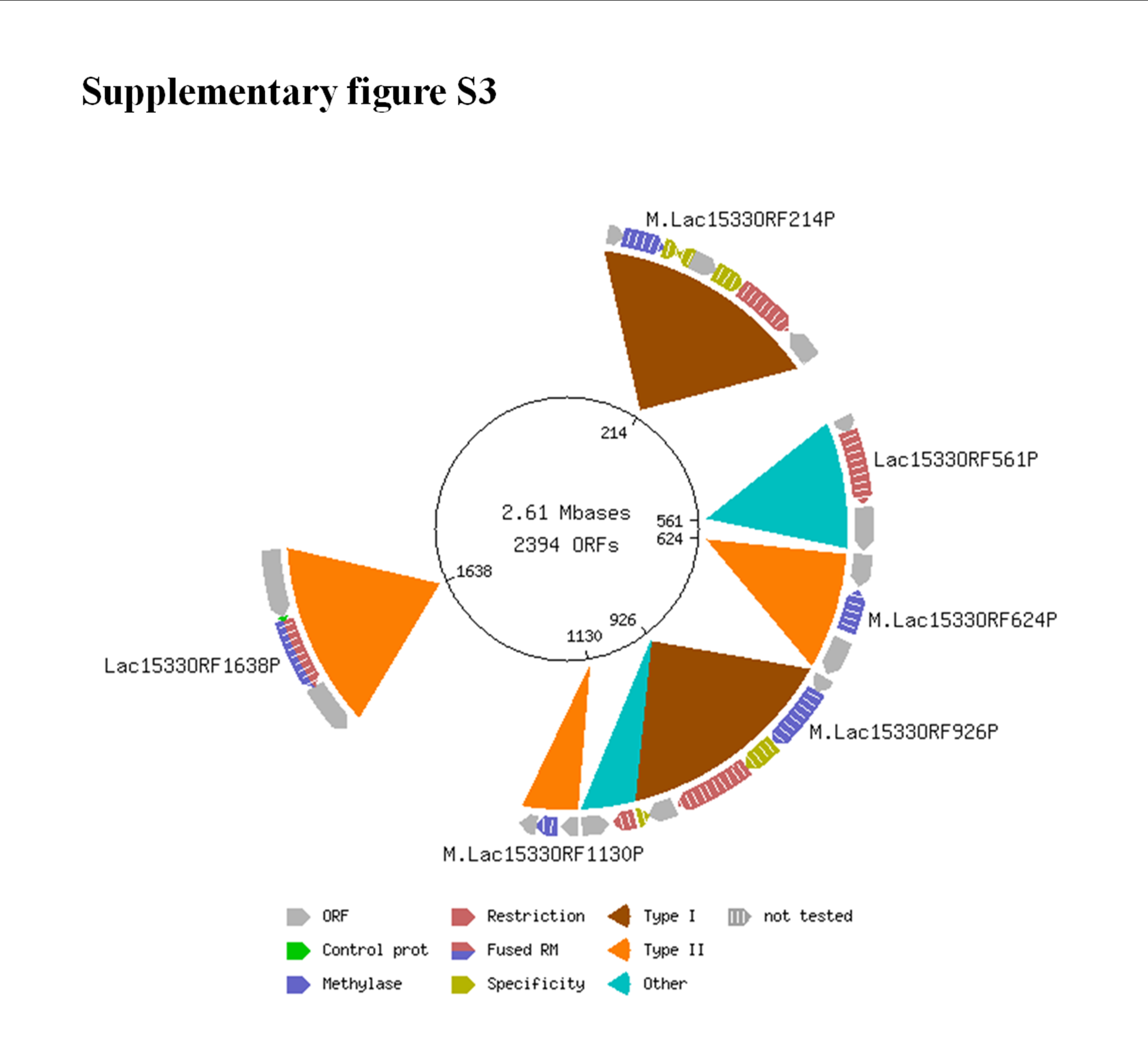

Supplement: FIGURE S3 — Circular map of the L. acidipiscis ACA-DC 1533 chromosome highlighting the predicted RM systems by the REBASE database. The symbols are color coded as indicated at the bottom of the figure. [file Image_3.tif]

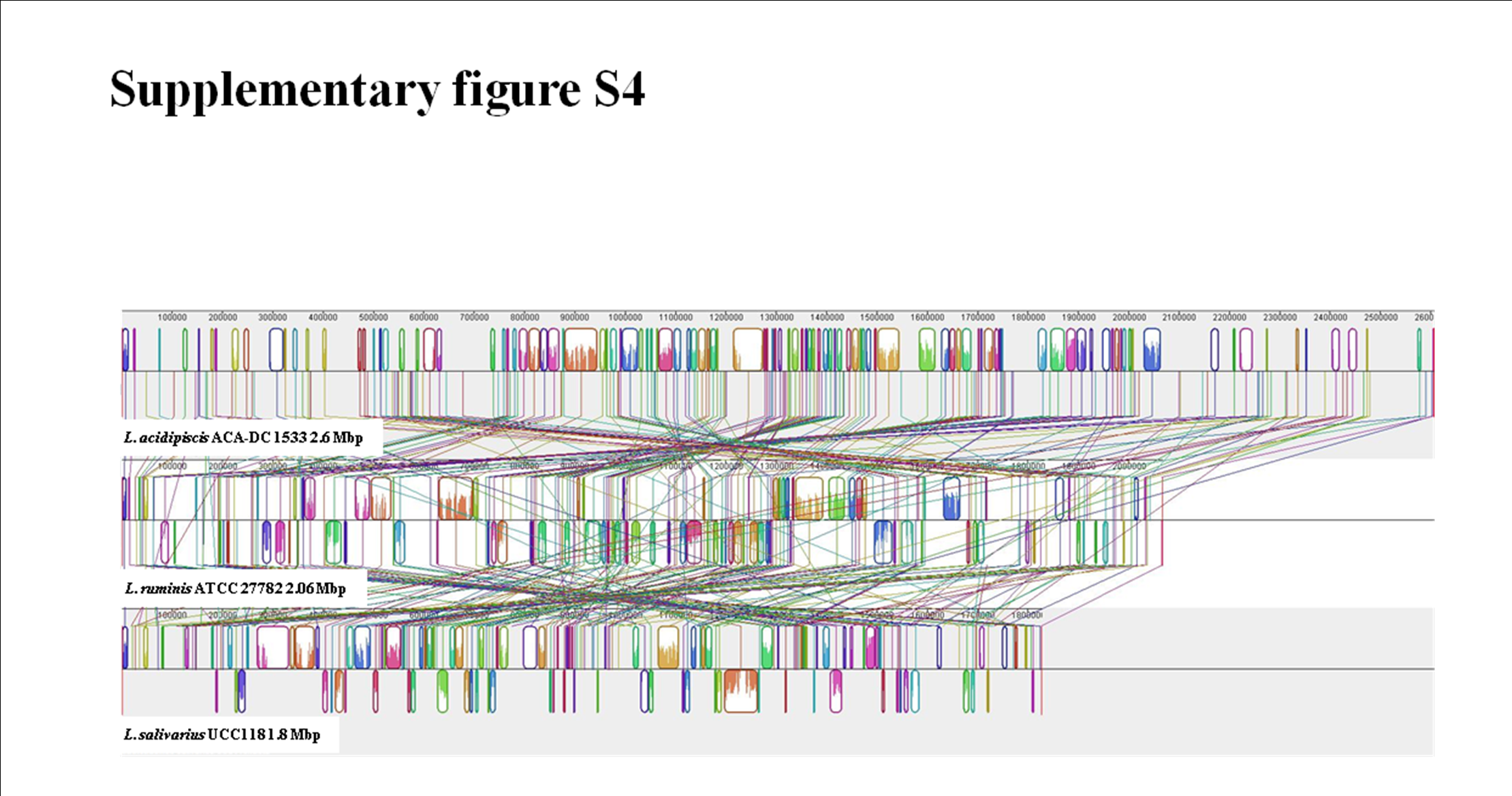

Supplement: FIGURE S4 — Chromosome alignments among the L. acidipiscis ACA-DC 1533, L. salivarius UCC118 and L. ruminis ATCC 27782 strains generated by progressiveMAUVE. Locally collinear blocks (LCBs) of conserved sequences are presented by the same color (white corresponds to the strain-specific regions). [file Image_4.tif]
